# Complete mitochondrial genome of *Iksookimia hugowolfeldi* and its phylogenetic position within genus *Iksookimia*

**DOI:** 10.1080/23802359.2020.1715292

**Published:** 2020-01-27

**Authors:** Philjae Kim, Jeong-Ho Han, Seung Lak An

**Affiliations:** a Research and Development Division, National Science Museum, Daejeon, Korea;; b Water Environmental Management Department, Korea Water Resources Corporation, Daejeon, Korea

**Keywords:** Korean loach species, complete mitogenome, *Iksookimia hugowolfeldi*, taxonomic relationship

## Abstract

In this study, the complete mitochondrial genome sequences of *Iksookimia hugowolfeldi*, Korean loach species, was determined using next-generation sequencing analysis. The complete mitogenome of *I*. *hugowolfeldi* has 16,634 bp in length and consists of 13 protein-coding genes (PCGs), 22 tRNAs, two rRNAs, and one control region (D-loop). Both gene orders and characteristics were exactly accord with mitochondrial genome of other species those belong to the family Cobitidae. Phylogenetic analysis revealed that the establishment of taxonomic relationship between *Iksookimia* and *Cobitis* has still uncompleted because of the not distinguished as monophyletic status.

Genus *Iksookimia* belonging to family Cobitidae of order Cypriniformes has reclassified from *Cobitis* (Nalbant 1993). *Iksookimia hugowolfeldi* is one of the *Iksookimia* and known to as a Korean indigenous species (Kim 2009). This species has been confused with *I. longicorpa* because of similar morphological characteristics. However, in 1993, Nalbant T.T. found Yeongsan river-dwelling *I. longicorpa* group has several differences in morphological features and reported them as a new species named *I. hugowolfeldi*. Since the genus *Iksookimia* newly erected, the molecular studies have not conducted yet, whereas many studies have done in entire Cobitidae (Kim et al. 2000; Šlechtová et al. 2008; Kwan 2015). Therefore, we provide the complete *I. hugowolfeldi* mitogenome sequences (MN756662) for contributing the establishment of evolutionary relationship of *Iksookimia*.

The specimen was collected in Suncheon, Korea (35°01′20.04″N, 127°20′43.03″E) on 19 August 2019 and identified based on the morphological studies (Kim 1997, 2009). Voucher specimens (NSMK-FI00003) and mitochondrial DNA (mt-DNA) sample (NSMK-DN00003) were deposited in storage and freezer (–80 °C) of Natural History Laboratory, National Science Museum (Daejeon, Korea), respectively. First, we isolated the mitochondria from caudal fin using Qproteome^®^ Mitochondria Isolation Kit (QIAGEN, Hilden, Germany). Then, we extracted DNA using DNeasy Blood & Tissue DNA isolation kit (QIAGEN). PCR product for next-generation sequencing (NGS) analysis was prepared using mitochondrial DNA by REPLI-g Mitochondrial DNA Kit (Qiagen). A genomic library was constructed using QIAseq FX single cell DNA library kit (QIAGEN) and used for NGS analysis in GnC Bio Co. (Daejeon, South Korea) followed by the Illumina Hi-Seq 2500 platform (San Diego, CA, USA).

The complete mitogenome of *I. hugowolfeldi* (MN756662) is composed 16,634 bp of nucleotide. The genome consists of 13 PCGs, 22 tRNA genes, two rRNA genes, and one control region (D-loop). The gene order and arrangement were completely accord with other Cobitidae species. For all PCGs, the most common initiation codon is ‘ATG,’ except for COI started with ‘GTG’; The most common termination codon is ‘TAA’ (ND1, COX1, COX2, ATP8, ATP6, ND4L, ND4, and ND6), then is ‘TAG’ (ND5). The rest of four PCGs (ND2, COX3, ND3, and CytB) have incomplete terminal codon ‘T––.’ The mitochondrial base composition is 30.3% A, 25.3% T, 28.5% C, and 15.9% G.

The dataset for molecular phylogenetic analysis included 13 PCGs of five *Iksookimia* species. One relative species, *Misgurnus mizolepis* (MF579258) belonging to family Cobitidae, was used as outgroup. The maximum-likelihood analysis was conducted using PhyML 3.1 with GTR + G model (Guindon et al. 2010). The bootstrap assembling was performed 1000 replication with rapid options.

In phylogenetic analysis, *I. hugowolfeldi* formed monophyletic clade. However, *Cobitis* and *Iksookimia* species were grouped with the state of undistinguished each genus (Figure 1). This result showed two genera have confusion in classification by the discordance between molecular relationship and morphological taxonomic status. Therefore, we suggested the taxonomical relationship between *Iksookimia* and *Cobitis* should be reestablished. Furthermore, we carefully supposed the consideration of *Iksookimia* as a synonym for *Cobitis* is necessary.

**Figure 1.**
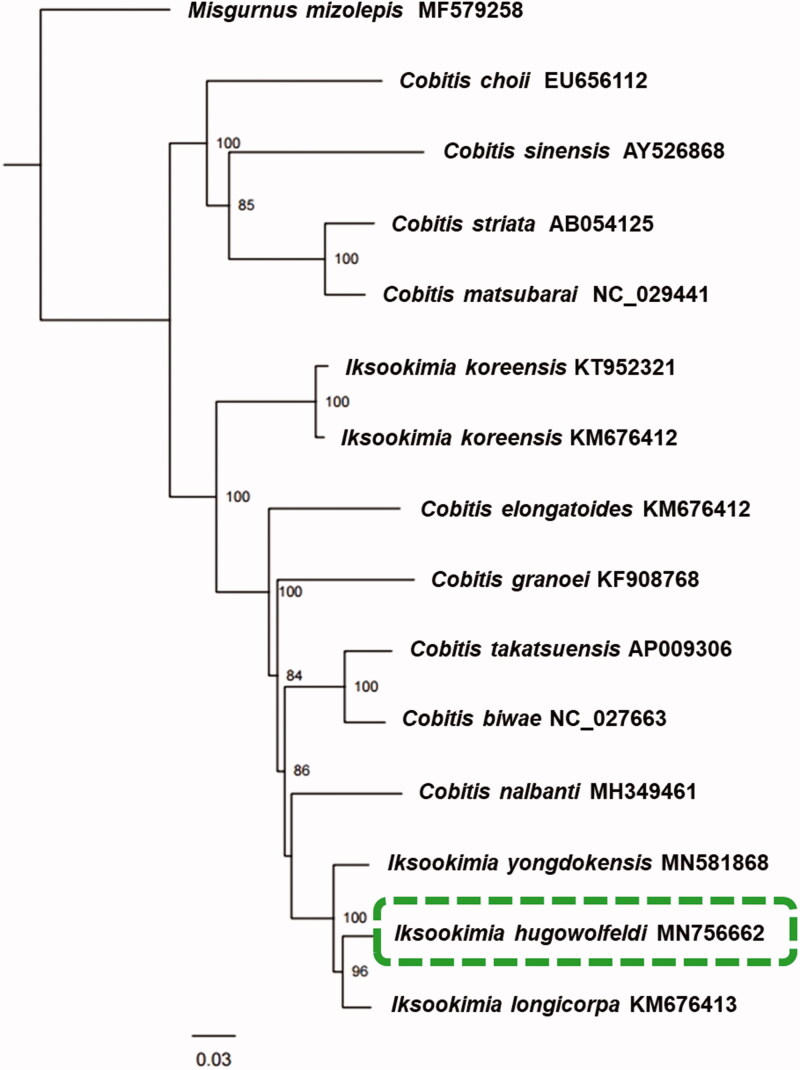
Phylogenetic tree inferred by maximum-likelihood using of 13 protein-coding genes of 15 mitochondrial genome of 14 cobitid species, including *I. hugowolfeldi* (MN756662) and one outgroup species belongs to family Cobitidae. Bootstrap support values based on 1000 replicates are displayed on each node as >70.
